# Depression, anxiety and their associated factors among patients with tuberculosis attending in Gondar city health facilities, North West Ethiopia

**DOI:** 10.1186/s12888-023-04573-7

**Published:** 2023-02-06

**Authors:** Solomon Assefa, Berhanu Boru, Daniel Ayelegne Gebeyehu, Bewuketu Terefe

**Affiliations:** 1 School of Nursing, Department of Medical Nursing, College of Medicine and Health Sciences, University of Gondar, Gondar, Ethiopia; 2 School of Nursing, Department of Community Health Nursing, College ofMedicine and Health Sciences, University of Gondar, 196 Gondar, Ethiopia

**Keywords:** Anxiety, Depression, Health facilities, Patients, Tuberculosis

## Abstract

**Introduction:**

Depression and anxiety are the most prevalent mental disorders in the general population and are expected to be the number one global burden of disease by the year 2030. They are also common comorbid conditions for patients with tuberculosis.

**Objective:**

This study aimed to assess the prevalence of symptoms of depression, and anxiety and their associated factors among patients with tuberculosis attending Gondar city health facilities.

**Methods:**

An institution-based cross-sectional study was conducted from September 01 to 30/2020. A census sampling technique was employed to select 390 patients. A structured interviewer-administered questionnaire was used to collect data, and a standardized hospital anxiety and depression scale was used to measure the symptoms of anxiety and depression. Data were entered in Epi-Info version 7 and analyzed using SPSS version 23. Binary and multivariable logistic regressions were computed to identify factors associated with the symptoms of depression and anxiety. P-value < 0.05 and adjusted odds ratios were used to declare the significance and strength of the association.

**Results:**

The overall prevalence of symptoms of depression and anxiety were found to be 35.8% with 95% CI (34.6, 36.6). Perceived stigma and duration of illness > 12 months were associated positively ([AOR = 3.60; 95% CI (2.74, 4.43)], and [AOR = 3.19; 95% CI (2.17, 4.19)]) for both depression and anxiety respectively. Separate analyses revealed that the prevalence of symptoms of depression was 55.9% (95% CI (51.0%, 60.3%) and was significantly associated with duration of illness 4–6 months and > 12 months (AOR = 1.21; 95% CI (1.17, 2.73)] and [AOR = 2.36; 95% CI (2.16, 3.79)], comorbid chronic disease (AOR = 0.12; 95% CI (0.08, 0.91)] and perceived stigma [AOR = 0. The prevalence of anxiety symptoms was 39.5 percent, with 95% confidence intervals of 34.6% and 44.6%, and it was significantly associated with comorbid chronic disease [AOR = 2.53; 95% CI (1.96, 6.32)] and perceived stigma [AOR = 3.31; 95% CI (1.22, 7.74)].

**Conclusion:**

The prevalence of symptoms of depression and anxiety was high. Duration of illness, comorbid chronic disease, and perceived stigma were significantly associated with symptoms of depression. Comorbid chronic disease and perceived stigma were significantly associated with symptoms of anxiety.

## Introduction

Tuberculosis (TB) is considered one of the deadliest transmissible diseases. It is a chronic infectious disease caused by Mycobacterium tuberculosis. According to the World Health Organization's 2020 report, a total of 1.5 million people died from TB in 2020 (including 214 000 people with HIV). TB is the 13th leading cause of death worldwide and the second leading infectious killer after COVID-19 [1]. Evidence suggests that more than 300 million people globally suffer from depression [2]. Similarly, anxiety was the sixth most prominent cause of non-fatal health loss and is thought to affect 264 million individuals worldwide. 2. Studies have indicated the comorbidity of depression and anxiety with chronic illnesses, especially TB, is very common [3, 4].

Depression is a common mental disorder characterized by a loss of interest or pleasure, guilt or low self-worth, disturbed sleep or appetite, low energy and poor concentration, insomnia or hypersomnia, and occasionally suicidal thoughts for at least two weeks [5]. Anxiety, on the other hand, is characterized by a vague, subjective, non-specific sense of uneasiness, apprehension, tension, excessive nervousness, fears, a sense of impending doom, irrational avoidance of objects or situations, and attack [6].

There is a high prevalence of anxiety and depression in TB patients. A study in Wolita Sodo, Ethiopia, discovered that 43.4 percent (181) and 41.5 percent (173) of TB patients had depression and anxiety, respectively [7]. Moreover, there are certain factors like poor socioeconomic condition, HIV/AIDS [8], treatment non-adherence [9], perceived stigma [10], poor social support [11], substance use [9], being female [12], comorbid chronic illness [13], phase of treatment [14], family history of mental illness [15], sputum smear at time of diagnosis [16], were significantly associated with anxiety and depression in patients with TB.

Depression and anxiety can become chronic or recurring, causing significant impairments in an individual's ability to carry out daily activities [17]. Moreover, they affect the quality of life, health care costs, and self-care. As a result of decreased resistance to infections, patients' adherence to TB treatment suffers, potentially increasing disease mortality [18].

Despite the high prevalence of tuberculosis in Ethiopia, data on the majority of depression and anxiety are scarce. As a result, this study aimed to assess the prevalence of depression and anxiety in patients with TB using a larger sample size and some factors. The study's findings will raise awareness of governmental and non-governmental health organizations working to prevent tuberculosis and related psychosocial problems.

## Methods and materials

### Study design, period, and area

A cross-sectional study was conducted in Gondar health facilities, in Northwest Ethiopia, from September 01 to September 30/2020. Gondar is located in the northwest part of Ethiopia in the Amhara region about 737 km from Addis Ababa (the capital city of Ethiopia), and 184 km from the regional state of Amhara [19]. According to the 2007 population and housing census report, the total population size of Gondar city was estimated to be 206,987, of whom 98,085 were men and 108,904 were women. The city has 21 kebele, one referral hospital, and eight governmental health centers. Each health center serves the people who live nearby. Since its inception in 1954, the University of Gondar's comprehensive and specialized hospital has provided a wide range of services. It is a teaching hospital serving more than five million people in the Gondar zone and neighboring zones. The hospital provides services to newly diagnosed tuberculosis patients. All health centers offer services for TB treatment.

## Populations

### Source and study population

All clients on treatment for tuberculosis in Gondar health facilities were considered a source population, and clients who were on treatment in Gondar health facilities during the study period were the study population.

### Inclusion and exclusion criteria

All TB patients over 18, who had been receiving antituberculosis treatment for at least two weeks, and who volunteered to participate were included in the study. In contrast, those who were seriously ill, unable to respond to the questions, or deaf were excluded.

### Sample size determination

The required sample size for this study was calculated using the single population proportion formula and considering a 95% confidence interval and a 5% margin of error. The prevalence of symptoms of depression p = 43.4% and Anxiety p = 41.5% were used from a previous study in which [8].

$$\mathrm n=\frac{\left(\frac{{\mathrm Z}_{\mathrm\alpha}}2\right)^2\;\mathrm P\left(1-\mathrm p\right)}{\mathrm d^2}$$

Where;

n = sample size.

P = 0.434 from the prevalence of depression symptoms conducted in Wolaita Sodo University Hospital and Sodo health center in southern Ethiopia (10).

Z_α/2_ = the standard normal value of Z at α/2 and (1- α) %, i.e.,1—0.05, 95% confidence level.

For α = 0.05 the Z_α/2_ = Z_0.025_ = 1.96,

d = Margin of error.

Therefore, at a 95% confidence level and 5% margin of error, p = 0.434** = **0.5, d = 5% = 0.05.

$$\mathrm n=\frac{\left(\frac{{\mathrm Z}_{\mathrm\alpha}}2\right)^2\;\mathrm P\left(1-\mathrm p\right)}{\mathrm d^2}$$

$$\mathrm n=\frac{\left(1.96\right)^2\;0.434\left(1-0.434\right)}{\left(0.05\right)^2}$$

*n* = 377 So, considering a 10% non-response rate, the sample size was 418**.**

The sample size for the second objective was calculated using statistically significant factors from a study on the prevalence and factors associated with the symptoms of anxiety and depression among tuberculosis patients in Gondar city health facilities in northwest Ethiopia. The final sample was 418. However, the report sheet from the health facilities shows that the total number of clients who are taking treatments and waiting for follow-up is 390. In other words, the total sample size calculated based on the two objectives was larger than the total number of clients. Therefore, all patients who had treatment follow-ups at all health facilities were included in the study.

### Sampling procedure

**Fig. 1:**
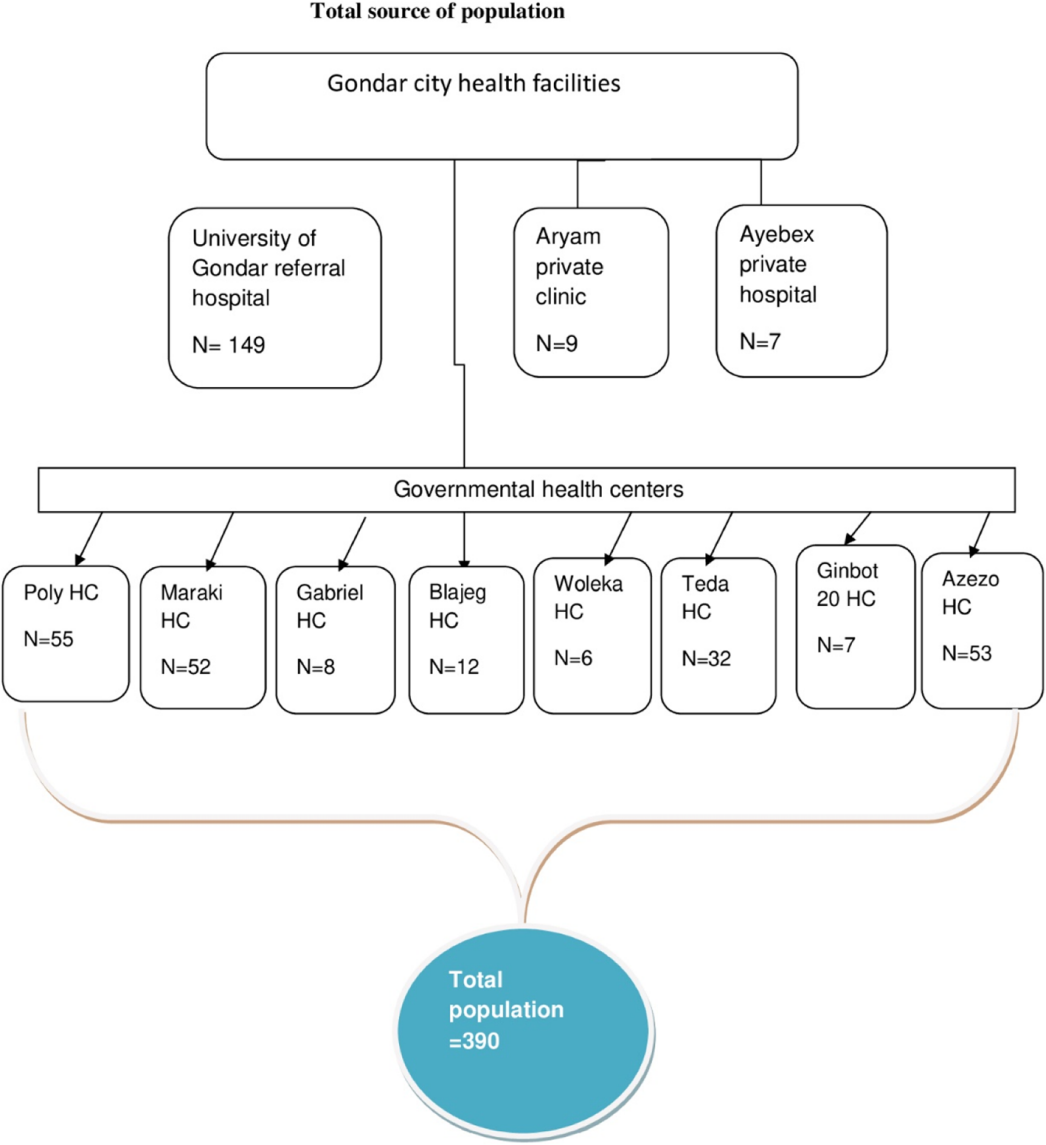
Schematic presentation of the sampling procedure to assess the prevalence and associated factors of symptoms of depression and anxiety among tuberculosis patients in Gondar city health facilities, 2020

The census recruited study participants in all eight health centers. The sampling procedure has been completed (Fig. 1).

### Data collection tool and procedure

Data were collected using a standardized questionnaire written in English and translated into Amharic by a medical and linguistic translator before being translated back into English after data collection to ensure that the content was retained. A structured interviewer-administered questionnaire was used to collect information from the study participants, and secondary data were obtained from patients' charts for clinical information. The questionnaire consisted of five parts, including socio-demographic characteristics, comorbid, behavioral, personal, and treatment-related factors. In Oslo, Norway, three-item social support scales were used to measure social support [20]. The items commonly used to assess social support have a sum score ranging from 3 to 14. It has three categories: poor social support (3–8), good social support (9–11), and strong social support (12–14). It has been considered that a score greater than or equal to 9 was considered good social support, whereas less than nine was considered poor social support. Data regarding perceived stigma was collected using a perceived TB stigma scale using a four-point Likert scale (strongly disagree, agree, disagree, and strongly agree). Participants were classified as having or not having perceived stigma, using the meaning of the stigma variable as a cut point.

Data were collected by eleven Nurses with diplomas, and two Psychiatry professionals with BSc degrees were assigned to supervise. Data collectors received thirteen hours of training regarding the tool, technique of interviewing, communication skills, techniques using possible TB preventive mechanisms, and checklist completion. A structured interviewer-administered questionnaire was used to collect information from the study participants, and secondary data were obtained from patients' charts for clinical information. The training was also given to data collectors regarding interviewing techniques, communication skills, and fitting of the checklist. The outcome variables, the symptoms of anxiety, and depression were measured using the 14-item questionnaire of the Hospital Anxiety and Depression Scale (HADS), which is widely used and validated in Ethiopia. Based on hospital anxiety and depression symptoms scale points, 0–7 were considered normal, and 8–21 were moderate to severe, which is to say that the cut-off point was eight.

### Data quality assurance

The translated Amharic version of an interview-administered questionnaire was distributed to the study participants. A pretest with 5% of the total sample size was done before collecting the actual data at Sanja primary hospital. Both the principal investigator and other authors checked the fitted questionnaire daily for completeness and neatness.

### Data processing and analysis

Data were coded and entered into Epi-info version 7, and statistical software for cleaning, storing, recording, and analyzing with SPSS version 23. Descriptive statistics like frequency, percentage, mean, median, and standard deviation were used to present the socio-demographic characteristics of the study participants. A binary logistic regression model was used to identify factors associated with the dependent variable with a *p*-value of < 0.25. In the multivariable logistic regression analysis, the significance level was declared at a *p*-value of 0.05 with a 95% confidence interval after they passed in the binary logistic regression model fitting with a criterion of *p*-value < 0.25. The Hosmer–Lemeshow goodness of fit test was used to assess model fitness.

## Results

### Socio-demographic characteristics of study participants

In this study, a total of 390 patients participated. The mean age of the respondents was 25.43 5.02 (SD) years. A majority (72.3%) of the respondents were 25–49 years old. More than half (55.9%) of the study participants were male. Regarding marital status, 193 (49.5%) of the participants were single. On the other hand, 156 (40%) of the respondents had attended primary school. Regarding occupation, 115 (29.5%) of the participants were self-employed, followed by housewives with 94 (24.1%). Most (80.5%) of the study participants settled in an urban area. More than half (53.1%) of respondents earn a monthly income of less than 500 Ethiopian birrs (Table 1).

### Treatment-related and behavioral characteristics of the respondents

Among the respondents, two hundred fifty-seven (65.7%) were diagnosed with pulmonary tuberculosis, of which 95.9% were on new TB treatment. Regarding the duration of illness, more than half (59.6) of the participants had a 3-month duration. More than half (57%) of the participants were in a continuous phase of TB treatment, followed by an intensive phase (43%). A majority (80%) of them had no chronic comorbid illness. Nearly half (52.4%) of the participants needed better social support. One hundred eighty-four (52.9%) respondents had no perceived stigma. More than half (57.5%) of the respondents did not currently use substances like tobacco, cigarettes, or alcohol (Table 2).

### Prevalence of symptoms of depression and anxiety among tuberculosis patients

With a 95% confidence interval of 34.6–36.6, the overall prevalence of depression and anxiety symptoms was 35.8%. The overall prevalence of depression and anxiety symptoms among tuberculosis patients was 55.9%, with a 95% confidence interval of 51.0%, 60.3%, and 39.5%, with a 95% confidence interval of 34.6% and 44.6%), respectively.

### Factors associated with symptoms of depression and anxiety among tuberculosis patients

Perceived stigma and illness duration > 12 months were positively associated with depression and anxiety symptoms (AOR = 3.60; 95% CI: 2.74, 4.43; and [AOR = 3.19; 95% CI: 2.17, 4.19]). In binary logistic regression analysis, however, the duration of illness, comorbid chronic diseases, social support, and perceived TB stigma were all significantly associated with the symptoms of anxiety. All the variables related to the bivariable logistic regression analysis were included in the multivariable regression analysis. In multivariable logistic regression analysis, factors significantly associated with depression and anxiety symptoms were duration of illness, comorbid chronic disease, and perceived TB stigma. Symptom of Anxiety was significantly associated with comorbid chronic disease in multivariable logistic regression analysis and perceived TB stigma was significantly associated with the symptoms of anxiety.

The odds of depression symptoms were 2.36 times higher among patients with a duration of illness more significant than 12 months compared to those with a duration of illness less than or equal to 3 and 4–6 months [AOR = 2.36; 95% CI (2.16, 3.79)]. Patients who did not have the comorbid chronic disease were 88% less likely to develop symptoms of depression than those who did [AOR = 0.12; 95% CI (0.08, 0.91)]. Similarly, patients who did not experience perceived TB stigma were 85% less likely to develop symptoms of depression than those who did [AOR = 0.15; 95% CI (0.09, 0.25)] (Table 3).

When compared to patients who did not have a comorbid chronic disease, the odds of the symptom of anxiety were nearly 2.5 times higher [AOR = 2.53; 95% CI (1.96, 6.32)]. Similarly, the odds of anxiety symptoms were almost three times higher in patients who had perceived TB stigma versus patients who did not have perceived TB stigma [AOR = 3.31; 95% CI [1.22, 7.74]] (Table 4).

## Discussion

The principal aim of this study was to assess the prevalence of symptoms of depression, anxiety, and their associated factors among tuberculosis patients attending Gondar health facilities. The findings of this study showed that the prevalence of symptoms of depression was 55.9%.

This finding was consistent with studies conducted in the Eastern part of Ethiopia (51.9%) and Brazil (54.6%) [21, 22]. On the other hand, it was lower than studies conducted in India (62%), Turkey (60.5%), and Cameroon (61.1%) [3, 23, 24]. The possible explanation for this difference might be due to variations in study design and the data collection instruments used. The study conducted in India used an observational design, and a nine-item Patient Health Questionnaire (PHQ-9), which has good psychometric properties compared to other validated instruments, was used to assess symptoms of depression. Another possible reason might be the socioeconomic and cultural differences between the previous and current studies. Nonetheless, the prevalence of depression symptoms in the current study was higher than in studies conducted in Addis Ababa, Ethiopia (31.1%), Wolaita Sodo Hospital, Ethiopia (43.4%), Jimma Hospital, Ethiopia (19.2%), Uganda (23.7%), Nigeria (45.5%), Tanzania (36%), and China (17.73%) [25–30]. This discrepancy might be due to differences in the sampling technique; the studies conducted in Wolayta Sodo and Addis Ababa used a systematic random sampling technique, whereas the current study used a census method. The difference in study design might be another possible reason for the variation, as an ongoing prospective study was used in Nigeria.

The findings of this study also showed that the prevalence of anxiety symptoms was 39.5%. This finding was consistent with a study conducted in Ethiopia (41.5%) [21]. However, it was lower than a study conducted in Ethiopia (65%) [30]. The possible justification for the difference might be the difference in the study setting and participants. The findings of this study were higher than studies conducted in Tanzania (31%), Turkey (26%), and China (18.13%) [3, 27, 29]. The difference might be due to a difference in study participants; the Tanzanian study was conducted among out-of-school adolescent girls and young women. Similarly, the study in China incorporates patients with pulmonary tuberculosis who were inpatients and newly diagnosed or undergoing treatment via directly observed therapy (DOT). But all patients on follow-up for tuberculosis treatment were included in the current study.

Duration of illness, comorbid chronic disease, and perceived TB stigma were significantly associated with the symptoms of depression. The odds of symptoms of depression were 1.21 and 2.36 times higher among patients with a duration of illness of 4–6 months and > 12 months compared to those with a duration of illness of less than or equal to three months, respectively. This finding was supported by studies conducted in Nigeria and Pakistan [25, 31]. The possible explanation for this might be that, as the duration of illness increases (chronic tuberculosis infection), patients may develop symptoms of depression due to related psychosocial stressors and general factors like weight loss and the severity of medical illness. Medically severe illnesses are intense stressors that affect body image, self-esteem, and the capacity to maintain family and social relationships [32, 33]).

Patients without comorbid chronic diseases were 88% less likely to develop symptoms of depression than those with comorbid chronic diseases. This finding was supported by studies conducted in Ethiopia, Uganda, Cameroon, and China [15, 26, 28, 30, 34]. This could be because being diagnosed with a comorbid chronic disease, specifically HIV, a life-long terminal disease associated with high levels of stigma, may also lead to high rates of mental disorder [35]. Similarly, patients without perceived TB stigma were 85% less likely to develop symptoms of depression than those who had perceived TB stigma. This finding was supported by studies conducted in Ethiopia, Turkey, India, and the USA [21, 36–38]. This might be because tuberculosis is a historically stigmatized disease; patients with perceived TB stigma stand as decreasing attributes that arise from social interaction. This might be blocked because of the patient's capacity to cope with the problem, leading to social isolation, sadness, self-stigmatization, and unemployment. As a result, this can easily lead to depression [39, 40]. Comorbid chronic disease and perceived TB stigma were significantly associated with anxiety. The odds of symptoms of anxiety were nearly 2.5 times higher among patients with the comorbid chronic disease compared to those who had no comorbid chronic disease. This finding was supported by studies conducted in Ethiopia, Uganda, Cameroon, and China [15, 23, 26, 28, 30]. This could be because patients with chronic comorbid diseases, such as HIV, are more likely to develop common mental disorders due to the stigma and discrimination they face in society [41]. Similarly, the odds of symptoms of anxiety were nearly three times higher among patients with a perceived TB stigma compared to patients with no perceived TB stigma. This finding was supported by studies conducted in Ethiopia, Turkey, India, and the USA [21, 36–38].

Perceived stigma is the worry that one will be devalued after a TB diagnosis. For a person with a TB diagnosis, this is the fear that the stigma against the person will be so bad that it affects treatment. It may delay people from returning for care or impact adherence to the prescribed drugs. Patients with perceived TB stigma isolate themselves from social interactions and develop anxiety. Patients with tuberculosis may experience symptoms of anxiety due to feelings of shame, embarrassment, or social isolation [41, 42].

### Strengths and limitations of the study

Because it includes total patients with TB, there are no more sampling-related errors. Compared to the sampling technique, this study gave more reliable, accurate data, and it was more suitable for these populations because it included all the study participants in the study area; however, since it was a health facility-based study, it might not be the correct representation of the community. The results may not be generalized to the community population. The accuracy and recall bias may be affected by later events and experiences because participants may not remember earlier events or experiences accurately or omit details. Furthermore, an interviewer bias could occur.

## Conclusion and recommendation

The prevalence of symptoms of depression and anxiety among tuberculosis patients was found to be high. Duration of illness, comorbid chronic disease, and perceived TB stigma were significantly associated with symptoms of depression. Comorbid chronic disease and perceived TB stigma were significantly associated with symptoms of anxiety.

Interventions for screening and controlling mental health (symptoms of depression and anxiety) are best intensified through multidisciplinary and holistic care for TB patients to identify patients who require further psychosocial assessment and support. Health facilities that are providing TB screening and treatment need to assess patients for the symptoms of depression and anxiety and provide intervention, giving more emphasis to patients with a long duration of illness and chronic comorbid disease. Health professionals working in all TB treatment centers can better assess patients for depression and anxiety and provide appropriate psychological and medical treatment since the prevalence of depression and anxiety is high. Finally, it is better to reduce the stigma associated with tuberculosis through the wide dissemination of information, education, and communication. Finally, for upcoming investigators to identify additional factors, mixed methods are suitable.

**Table 1:** Socio-demographic characteristics of tuberculosis patients attending in Gondar city health facilities, Northwest Ethiopia, 2020 (*n*= 390)

Variables	frequency	Percent (%)
Age (Years)		
18-24	85	21.8%
25-49	282	72.3%
> = 50	23	5.9%
Sex		
Male	218	55.9%
Female	172	44.1%
Marital status		
Single	193	49.5%
Married	159	40.8%
Divorced	34	8.7%
Widowed	4	1%
Educational status		
Can’t read and write	93	23.8%
Primary school (1-8)	156	40%
Secondary school (9-12)	101	25.8%
Diploma and above	38	9.7%
Other specify^a^	2	0.5%
Occupational status		
Civil servant	28	7.2%
Farmer	44	11.3%
Self-employed	115	29.5%
Merchant	35	9.0%
Student	61	15.6%
Housewife	94	24.1%
Other specify^b^	13	3.3%
Residence		
Urban	314	80.3%
Rural	75	19.2%
Monthly income		
< 500ETB	207	53.1%
500ETB	183	46.9%

^a^traditionally and religiously educated participants, ^b^daily labor, fired from work

**Table 2:** Treatment-related and behavioral characteristics of tuberculosis patients attending in Gondar city health facilities, 2020 (*n*= 390)

Variables	Frequency	Percent (%)
Classification		
Pulmonary TB	257	65.7%
Extra-pulmonary TB	134	34.3%
Category of treatment		
New	375	95.9%
Return after default	11	2.8%
Relapse/treatment after failure	5	1.3%
Duration of illness		
<= 3 months	233	59.6%
4-6 months	104	26.6%
>12 months	54	13.8%
Phase of treatment		
Intensive phase	168	43%
Continuous phase	223	57%
Co-morbid chronic illness		
Yes	78	20%
No	312	80%
Social support		
Good	186	47.6%
Poor	205	52.4%
Perceived TB Stigma		
Yes	207	47.1%
No	184	52.9%
Substance use (alcohol, chat, and cigarette)		
Yes	165	42.2%
No	225	57.5%

**Table 3:** Bivariable and multivariable logistic regression analysis of factors associated with symptom of depression among tuberculosis patients attending in Gondar city health facilities, 2020 (*n*= 390)

Variables	Depression		OR with 95% CI		*P*-value
	Yes	No	Crude	Adjusted	for AOR
Duration of illness					
< = 3 months	142	90	1	1	
4-6 months	57	47	1.30 (1.12, 2.64)	1.21 (1.17, 2.73)*	0.005
> 12 months	19	35	2.91(1.23, 4.88)	2.36 (2.16, 3.79)*	0.012
Co-morbid chronic disease					
Yes	20	59	0.19 (0.06, 0.67)	0.12 (0.08, 0.91) *	< 0.001
No	198	113	1	1	
Social support					
Poor	102	102	0.60 (0.11, 0.78)	0.46 (0.38, 1.56)	0.06
Good	116	70	1	1	
Perceived TB stigma					
Yes	77	130	0.18 (0.11, 0.28)	0.15 (0.09, 0.25) *	< 0.001
No	141	42	1	1	

*Statistically significant at *p*-value <0.05

**Table 4:** Bivariate and multivariable logistic regression analysis of factors associated with symptoms of anxiety among tuberculosis patients attending Gondar city health facilities, 2020 (*n* = 390)

Variables	Anxiety		OR with 95% CI		*P*-value
	Yes	No	Crude	Adjusted	for AOR
Comorbid chronic disease					
Yes	55	24	4.91 (2.97, 8.12)	2.53(1.96, 6.32)*	0.001
No	99	212	1	1	
Social support Poor	91	113	1.57 (1.04, 2.37)	1.41 (0.88, 2.25)	0.205
Good	63	123	1	1	
Perceived TB stigma					
Yes	117	90	5.13 (3.26,8.07)	3.31 (1.22, 7.74)*	< 0.001
No	37	146	1	1	

^*^ Statistically significant at *p*-value < 0.05

## Data Availability

Data supporting the findings in this paper are available upon reasonable request from the corresponding author, and the summary data are available in the main document.
